# On the Ferroelectric to Paraelectric Structural Transition of BaTiO_3_ Micro-/Nanoparticles and Their Epoxy Nanocomposites

**DOI:** 10.3390/molecules25112686

**Published:** 2020-06-09

**Authors:** Georgia C. Manika, Konstantinos S. Andrikopoulos, Georgios C. Psarras

**Affiliations:** 1 Smart Materials & Nanodielectrics Laboratory, Department of Materials Science, University of Patras, 26504 Patras, Greece; georgia.manika@chalmers.se; 2 Department of Industrial and Materials Science, Division of Engineering Materials, Chalmers University of Technology, SE-41296 Gothenburg, Sweden; 3 Foundation for Research & Technology-Hellas (FORTH), Institute of Chemical Engineering Sciences (ICE-HT), 26504 Patras, Greece; candrik@iceht.forth.gr

**Keywords:** BaTiO_3_ particles, polarazition, ferroelectric to paraelectric transition, Curie temperature, Raman spectroscopy, dielectric response

## Abstract

BaTiO_3_ is one of the most widely used ceramic components in capacitor formulation due to its exceptional ferroelectric properties. The structural transition from the ferroelectric tetragonal to the paraelectric cubic phase has been studied in both nano- and micro-BaTiO_3_ particles. Several experimental techniques were employed for characterization purposes (X-ray diffraction-XRD, laser Raman spectroscopy-LRS, differential scanning calorimetry-DSC and broadband dielectric spectroscopy-BDS). All gave evidence for the structural transition from the polar tetragonal to the non-polar cubic phase in both nano- and micro-BaTiO_3_ particles. Variation of Full Width at Half Maximum (FWHM) with temperature in XRD peaks was employed for the determination of the critical Curie temperature (T_c_). In micro-BaTiO_3_ particles (T_c_) lies close to 120 °C, while in nanoparticles the transition is complicated due to the influence of particles’ size. Below (T_c_) both phases co-exist in nanoparticles. (T_c_) was also determined via the temperature dependence of FWHM and found to be 115 °C. DSC, LRS and BDS provided direct results, indicating the transition in both nano- and micro-BaTiO_3_ particles. Finally, the 15 parts per hundred resin per weight (phr) BaTiO_3_/epoxy nanocomposite revealed also the transition through the peak formation at approximately 130 °C in the variation of FWHM with temperature. The present work introduces, for the first time, a qualitative tool for the determination and study of the ferroelectric to paraelectric structural transition in both nano- and micro-ferroelectric particles and in their nanocomposites. Moreover, its novelty lies on the effect of crystals’ size upon the ferroelectric to the paraelectric phase transition and its influence on physical properties of BaTiO_3_.

## 1. Introduction

Barium titanate (BaTiO_3_, BT) was the first ceramic in which the ferroelectric behaviour was observed and it has a typical perovskite structure (ABO_3_). BT has been widely used in many electronic applications due to its excellent ferroelectric properties, exhibiting a high dielectric constant and low dielectric loss. The importance of ferroelectricity in materials’ performance is reflected in high-tech applications, such as electronic and microwave devices, energy storage systems, sensors, supercapacitors, etc. [1,2,3]. An additional significant factor for BT’s high applicability is the chemical and mechanical stability along with its non-toxicity. Specifically, BT has been utilized in multilayer ceramic capacitors (MLCC), dynamic random-access memory capacitors and electrooptical switching elements in communication systems [4,5,6]. Bulk BT is polymorphic occurring in R3m rhombohedral (r), Amm2 orthorhombic (o), P4mm tetragonal (t) and a Pm3m cubic (c) crystal phase according to its temperature. Curie or critical temperature defines the transition, upon heating, to cubic (paraelectric phase). Below the Curie temperature, T_c_ BT exhibits spontaneous polarization which can be reversed by the application of an electric field. As a displacive and order-disorder ferroelectric oxide, elementary dipoles of BT in the paraelectric phase, either vanish or their statistical average is zero. The c phase occurs at temperatures higher of the Curie point, T_c_, (120 °C). The other phases occur successively upon cooling. Specifically, BT undergoes a sequence of structural transitions from c→t at 120 °C, t→o at 5 °C and o→r at −90 °C. Each of these structures can be considered as a small distortion of the cubic unit cell. In the cubic phase the Ba^+2^ cations occupy the corners of the cube, with the body center position occupied by a single Ti^+4^ cation. The six face centered positions are occupied by the O^−2^ ions. Therefore, the BT lattice can be regarded as being constructed from TiO_6_ octahedra. The distortion due to elongation of the unit cell along an edge [001] leads to t-BT, along a face diagonal [011] leads to the o-BT, or along a body diagonal [111] leads to the r-BT. In the case of the orthorhombic structure there is a large uncertainty of its structural parameters, resulting in the complexity of this structure. In the three non-centrosymmetric structures the displacements of Ti^+4^ and O^−2^ as well as the Ti^+4^ -O^−2^ and O^−2^–O^−2^ distances play a major role in the polymorphic transformation and the ferroelectric properties [4,7,8].

Several studies [9,10,11,12,13,14,15,16], indicate that the physical properties of BT powder are critically affected by the particle size. For instance, the dielectric constant of BT is strongly size dependent and reaches a maximum value at intermediate crystallite size (0.8 µm), being attributed to the contributions of domain wall displacements. As the size decreases, the ferroelectricity gradually reduces and the dielectric constant is progressively suppressed, because of the intrinsic grain size effect and the dilution effect of the non-ferroelectric grain boundary layers. In addition, the decrement of crystalline size results in reducing of the tetragonality distortion. Several models [10,11,12] have been proposed to explain this behaviour. The surface layer model supports that the transition occurs within the particle in an outer cubic surface layer of a fixed thickness to a tetragonal core, with a variation of tetragonalities among these regions. So, as the particle size decreases the influence of the tetragonal core diminishes. With decreasing size, the phase transition assumes a more diffuse character and display a lower transition enthalpy. Begg et al. [10] reported that powders over 0.27 µm in size are of the tetragonal phase, while powders under 0.19 µm in size are of the cubic phase at room temperature. Furthermore, Li and Shih [11], reported that 80 nm BT nanopowder exhibits tetragonal phase and powder of 56–80 nm in size have a mixed type of both tetragonal and cubic phases and only powder under 56 nm have cubic phase. All these findings suggest that the study of BT transitions is a very complicated issue and have attracted a lot of scientific interest though all these years [17,18]. The main motivation of this research work is the establishment of a method which can successfully determine the Curie temperature of the tetragonal ferroelectric to the cubic paraelectric phase transition. Furthermore, the present study aims to investigate the effect of crystal size upon this transition and the determination of the presence of ferroelectric phase, below T_c_ in nano-BT crystals. Since the various crystal structures (orthorhombic, tetragonal and cubic) of BT exhibit different dielectric behaviour, the determination of the critical temperature is of paramount importance in terms of material’s applicability. The particle size plays also a key role, since T_c_ appears to be size dependent. An additional significant aspect of this work is the study of the t→c transition in the case of composite materials. The prominent advantage of composite materials is the ability to adjust their properties by varying the type and the fractions of the constituents. For instance, the epoxy/ceramic composites can exploit in a single materials system the advantages of the polymer matrix (i.e., light weighting, thermomechanical stability, flexibility, corrosion resistance and high dielectric breakdown strength) and the BT particles (i.e., enhanced dielectric performance, thermally varying polarization and functionality). This type of composites can be widely used in electronic industry, such as interlayer capacitors, adhesive capacitive layers, thermistors, energy storing devices, printed wiring boards, etc. To the best of our knowledge this is the first time where such an approach is presented.

In this work, the structural transitions in both micro- and nano- (<100 nm) BT particles is studied, by employing several experimental techniques for validation purposes. A method for the qualitative identification of (T_c_) is proposed and tested in micro- and nano-BT particles. The crystal structure of BT is typically observed via X-ray diffraction (XRD) patterns and it appears to undergo transition from t→c phase approximately at 120 °C. However, XRD seems not to be very sensitive to transitions involving oxygen/titanium displacements. For this reason, laser Raman spectroscopy (LRS) was employed, since it can detect local lattice distortions and crystallographic defects at the molecular level. Moreover, differential scanning calorimetry (DSC), and broadband dielectric spectroscopy (BDS) gave evidence of the structural transition in BT micro- and nanoparticles. According to the best of our knowledge, there is lack of studies, providing a qualitative identification tool for this transition along with its validation by means of all these experimental techniques. Finally, a preliminary study of the t→c transition in the case of nanocomposite systems was also performed. Specifically, a nanocomposite system consisting of an epoxy resin and nano-BT particles has been prepared and the structural transition of embedded nano-BT particles was determined along with their T_c_.

## 2. Materials and Methods

### 2.1. Materials

**Figure 1:**
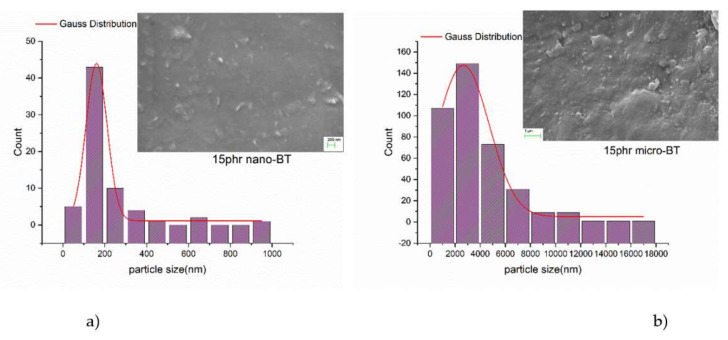
The particles’ size dispersion along with the Gaussian distribution function for (**a**) 15phr micro-BT and (**b**) 15 phr nano-BT/epoxy composite. Insets present representative SEM images.

The nano- and micro-BT particles were purchased by Sigma Aldrich (St. Louis, Missouri, USA) and as cited, the nanoparticles’ diameter was less than 100 nm, while in microparticles the mean diameter was 2 µm. The nanocomposite was prepared by employing commercially available materials. The polymer matrix was prepared using a low viscosity epoxy resin (ER) (bisphenol-A) with the trade name Epoxol 2004A, and a slow reacting cycloaliphatic amine as curing agent with the trade name Epoxol 2004B. Both reactants were provided by Neotex S.A. (Athens, Greece). The fabrication procedure was the following: (i) ER was mixed with curing agent at a 2:1 (w/w) ratio, (ii) as the matrix was in the liquid state BT nanoparticles were added, (iii) stirring at a slow rate under ultasonication was conducted in order to avoid the formation of clusters, and (iv) the homogenized mixtures were poured to moulds and the curing process took place for a week at ambient, followed by post-curing at 100 °C for 4 h. The filler content of the prepared nanocomposite sample, in parts per hundred resin (phr) per weight, was 15 phr. In order to investigate the size of both nano- and micro-particles, SEM images of cryo-fractured surfaces from micro- and nano-composites were examined. The size dispersion of the particles was determined by employing the Image J software (Figure 1a,b).

It was found that there is a wide dispersion of sizes with the major peaks to lie in 2–4 µm and 100–200 nm ranges in micro- and nanoparticles, respectively. In addition, the application of the Gaussian distribution provided indication that the average particle sizes are 160 nm and 2.6 µm, respectively, for nano- and microparticles. The obtained results are in good agreement with the values reported by Sigma Aldrich. The slight increase of the mean size of nanoparticles, can be attributed to the existence of limited small clusters within the composite specimen.

### 2.2. Methods

#### 2.2.1. X-ray Diffraction (XRD)

For the XRD experiments an AXS D8 Advance instrument (Bruker, Billerica, Massachusetts, USA) with Bragg-Bretano geometry was employed. A LynxEye detector and Cu Ka spectral line (λ = 1.54062 Å) was used as incident radiator. Scan mode was continuous, the step was 0.02° (2θ) and the scan speed was varied from 0.5 to 10 s/step. The source slit was 0.6 mm while the voltage and current were set at 40 kV and 40 mA, respectively. XRD spectra were recorded in the temperature range 30–160 °C by using a thermal chamber. The sample temperature was stabilized for 5 min before starting the measurements.

#### 2.2.2. Scanning Electron Microscopy (SEM)

The particle size dispersion was investigated by means of SEM images collected via an EVO MA 10 apparatus (Carl Zeiss, Oberkochen, Germany).

#### 2.2.3. Differential Scanning Calorimetry (DSC)

DSC measurements were conducted by a TA Q200 device (TA Instruments, New Castle, DE, USA). The scan rate operation was varied from 2 to 50 °C/min and the samples were placed in an aluminum crucible, while an empty one was used as reference. Temperature was ranging from 30 °C to 200 °C.

#### 2.2.4. Laser Raman Spectroscopy (LRS)

Raman experiments were held on a HR-800 JY UV-Vis Raman system (Horiba Scientific, Jobin Yvon, Villeneuve d’Ascq, France), where excitation was produced by an air cooled He-Cd laser operating at 441.6 nm. The laser beam power, measured on the sample, was 0.9 mW. The backscattering configuration was selected employing a 50 × (NA = 0.55) microscope objective. The scattered beam was directed to the entrance slit of the single monochromator after passing through the appropriate edge filter. The system was equipped with a LN_2_-cooled 2D-CCD detector. The sample heating was performed by an external hot stage with a heating rate of 20 °C/min. The temperature varied from 24 °C to 250 °C.

#### 2.2.5. Broadband Dielectric Spectroscopy (BDS)

For the dielectric characterization BDS was employed in the frequency range from 0.1 Hz to 10 MHz, using an Alpha-N frequency response analyzer, supplied by Novocontrol Technologies GmbH & Co. KG (Montabaur, Germany). The applied voltage amplitude was constant at 1V, while the temperature was controlled by Novotherm system with ±0.1 °C accuracy. The dielectric cell was a two terminal BDS 1308 system in the case of BT powders, and a BDS 1200 for the nanocomposite. Thermal controller and dielectric cells were all supplied by Novocontrol. The samples were placed between the gold-plated metal electrodes and isothermal frequency scans were conducted from ambient to 160 °C with a temperature step of 5 °C/min. Data acquisition was performed automatically in real time via the windeta software.

## 3. Results and Discussion

### 3.1. Micro BaTiO_3_ Particles

**Figure 2:**
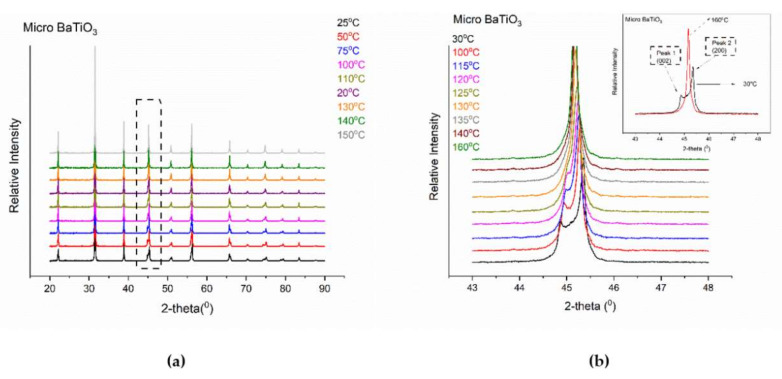
XRD patterns of micro-BT particles performed under the following experimental conditions: (**a**) increment = 0.02^°^ and scan speed = 0.5s and (**b**) increment = 0.01° and scan speed = 10 s. Inset shows the XRD patterns at 30 °C and 160 °C respectively.

XRD patterns of micro-BT particles were selected in the temperature range of 25–160 °C, for recording the t→c transition. (Figure 2a,b) represent the collected patterns in fast and slow scanning conditions.

It is well documented [19,20,21] that, below T_c_, micro-BT particles are in the tetragonal phase, giving two peaks in the range of 44–46° of 2-theta spectra, corresponding to the (002) and (200) reflections. On the other hand, above T_c_, the cubic lattice gives only one peak (200) in the same range. It is apparent from Figure 2b that micro-BT pattern at 30 °C forms two peaks, whereas in the XRD pattern of the same particles at 160 °C appears a single peak (inset of Figure 2b). Thus, the XRD profile of micro-BT particles at 30 °C indicates the tetragonal phase (P4mm), while at 160 °C represents the fingerprint of the cubic phase (Pm3m). The space groups were also confirmed through crystallographic analysis.

**Figure 3:**
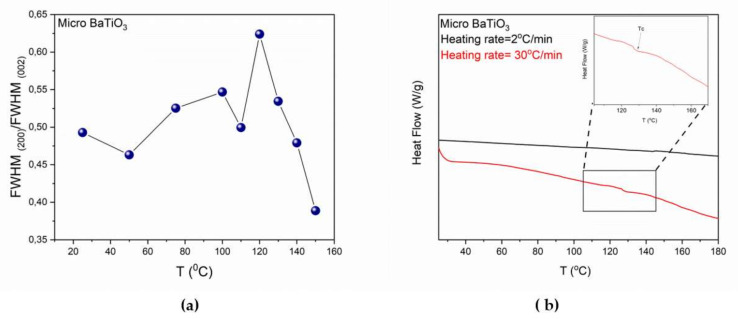
(**a**) FWHM_(200)_/FWHM_(002)_ as a function of temperature for micro-BT particles and (**b**) DSC thermograph of micro-BT particles varying the heating rate, endo down. Lines in Figure 2a are visual aid.

By performing deconvolution in all XRD patterns, the ratio of Full Width at Half Maximum (FWHM) of the two peaks, (200) upon (002), as a function of temperature was calculated and is presented in Figure 3a. Details for the deconvolution process and representative examples are given in the Supplementary Materials (Figure S1). The formation of a single peak, at temperatures higher than 120 °C, can be considered as a qualitative indicator of the t→c transition. In addition, thermodynamic theory has been applied for studying the transition [22]. First order transition, such as the one undergone by BT, between ferroelectric to paraelectric phase is characterized by a discontinuous change of the polarization at the transition temperature. This discontinuous change of the polarization is related with the intense peak in Figure 3a. The disorder to order transition refers to a rearrangement of the atoms’ position, which leads to a variation of polarization.

From thermodynamic point of view, the Gibbs free energy function takes the form of Equation (1) [22,23]:

(1) $$G\left(P,T\right)=G_{0}+\frac{\alpha }{2}P^{2}+\frac{b}{4}P^{4}+\frac{c}{6}P^{6}$$

where *G*_0_ represents the Gibbs function under unstressed and unpolarized conditions, *P* is the polarization, and parameters a, b and c are temperature dependent coefficients. In the vicinity of critical temperature, a can be approximated as a linear function of temperature [22,23] according to Equation (2):

(2) $$\alpha =\alpha_{0}\left(T-T_{C}\right),$$

where $\alpha_{0}$ is a positive constant [22]. For first order transitions coefficient b is considered as negative. In order to find the values of P for which G attains the lowest value (minimum free energy) Equation (1) is differentiated with respect to polarization, resulting in Equation (3):

(3) $$\frac{\partial G}{\partial P}=\alpha_{0}\left(T-T_{C}\right)P-\left|b\right|P^{3}+cP^{5}$$

It can be easily found that the solutions of Equation (3), at $T=T_{C}$, are: P=0 and $P=\pm {\left(\frac{\left|b\right|}{c}\right)}^{1/2}$ implying that polarization changes discontinuously at critical temperature [23]. In the first order transition P acquires a non-zero value immediately below $T_{C}$ [24].

Furthermore, DSC studies were also performed in micro-BT particles. Figure 3b shows DSC thermographs using two different heating rates. It is evident that only at a high heating rate the transition was recorded as a step-like variation of the heat capacity. Thermographs with low heating rate could not show the transition due to the quasi-static thermal condition of the sample. According to Equation (4) the heat flow signal in DSC consists of a function of heating rate, while the other component is a function of absolute temperature and time:

(4) $$\frac{dq}{dt}=C_{p}\frac{dT}{dt}+f\left(T,t\right)$$

where $\frac{dq}{dt}$ is the differential heat flow rate, $C_{p}$ is the heat capacity, $\frac{dT}{dt}$ is the heat rate and $f\left(T,t\right)$ is the kinetic heat flow. This equation indicates the significant influence of the heating rate on the total heat flow [25]. Moreover, at high heating rate sensitivity is increased in heat capacity measurements [25]. In the case of micro-BT, the high heating rate allows recording the transition as opposed to slow heating rate, because of the discontinuous change of heat capacity at *T_C_* [26,27,28].

**Figure 4:**
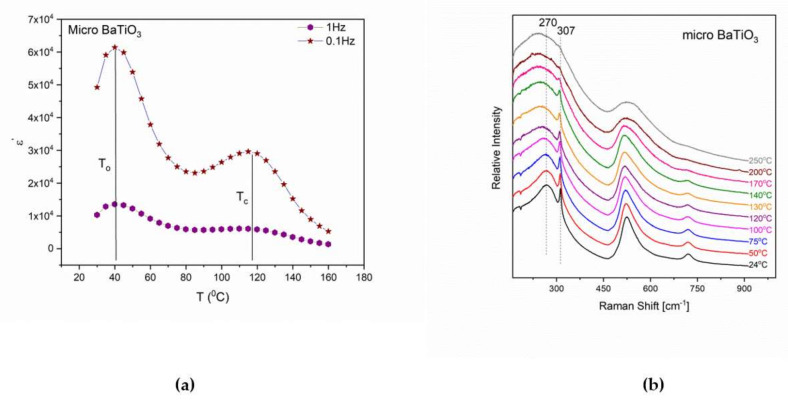
(**a**) Real part of dielectric permittivity as a function of temperature at 0.1 Hz and 1 Hz for micro-BT particles, and (**b**) Raman spectra for micro-BT particles in the temperature range from 24 °C to 250 °C.

Since the t→c transition is related to the variation of polarization, it can be monitored via dielectric spectra. Figure 4a depicts the variation of the real part of dielectric permittivity upon temperature, and the recorded peaks are directly related to the structural transitions of the micro-BT particles.

Figure 4a shows that, ε’ forms a broad peak, which is related to the t→c transition and lies between 110 °C and 120 °C. The broadening of this peak as well as its shift to lower temperature could probably be related to the ceramic (polycrystalline) nature of the particles, to the thermally agitated dynamics of the dipolar effects and to the inhomogeneous distribution of the time-varying electric field. Furthermore, an intense peak is observed at lower temperatures, $T_{o}\;=$ 40 °C, which corresponds to the o→t transition of BT and its location is in agreement with a previous research study [29]. In the literature it is reported that this transition occurs at lower temperatures (~ −4 °C) under DC conditions. However, the appearance of the peak at 40 °C is related to the frequency-temperature superposition, acting under the applied AC electric field, which finally shifts the transition temperature to higher values.

Raman spectra of micro-BT at various temperatures are depicted in Figure 4b. The Raman spectrum at 24 °C is characterized by vibrations and distortions indicative of the polar tetragonal phase. Tetragonal BT has 10 Raman active modes. When splitting of transverse and longitudinal optical modes, as well as splitting due to different polarizability in each unit cell, it is reported to have 18 first order Raman active optical phonons [30]. Bands at 307 cm^−1^, 520 cm^−1^ and 718 cm^−1^ are assigned to the tetragonal phase. Specifically, the peak at 307 cm^−1^ corresponds to the Ti-O vibration in the planed form of TiO_4_, which reduces its intensity as the temperature increases, providing an indication that the tetragonal phase transforms to the cubic. Although symmetry demands that no Raman active optical phonons should be present in cubic BT, Figure 4b depicts Raman active modes at 260 cm^−1^ and 530 cm^−1^. These peaks are associated with the atomic fluctuations of the tetragonal structure of the crystal [10,31,32,33]. Figure 4b indicates that above T_c_ the peaks owning to the tetragonal structure remain and only at much higher temperatures they are eliminated. This finding suggests that there is a range of tetragonalities distortions (variation of the c/a dimensional aspect ratio of the unit cell) in the powder crystals sample, resulting in this wide temperature interval of the transition [34].

### 3.2. Nanoparticles

**Figure 5:**
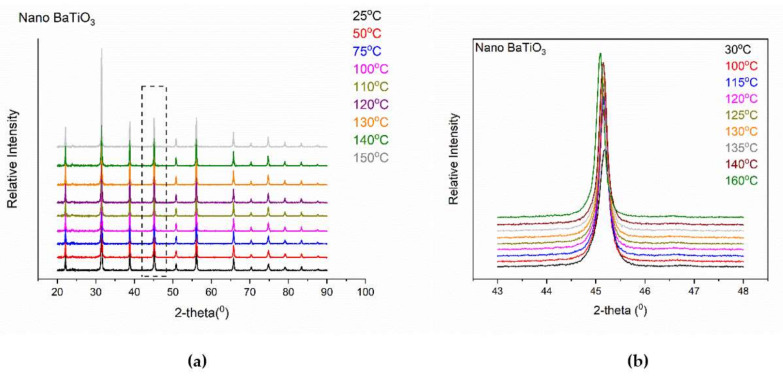
XRD patterns of nano-BT particles, at various temperatures, performed under the following experimental conditions: (**a**) increment = 0.02° and scan speed = 0.5s and (**b**) increment = 0.01° and scan speed = 10s.

The t→c transition concerned a lot of experimental and theoretical studies demonstrating that this structural transition is size dependent, with the ferroelectric phase becoming unstable at room temperature when particle’s dimensions decrease [7,13,35,36,37,38]. Figure 5 presents the XRD patterns of nano-BT particles in a wide temperature range for slow and fast scanning conditions.

**Figure 6:**
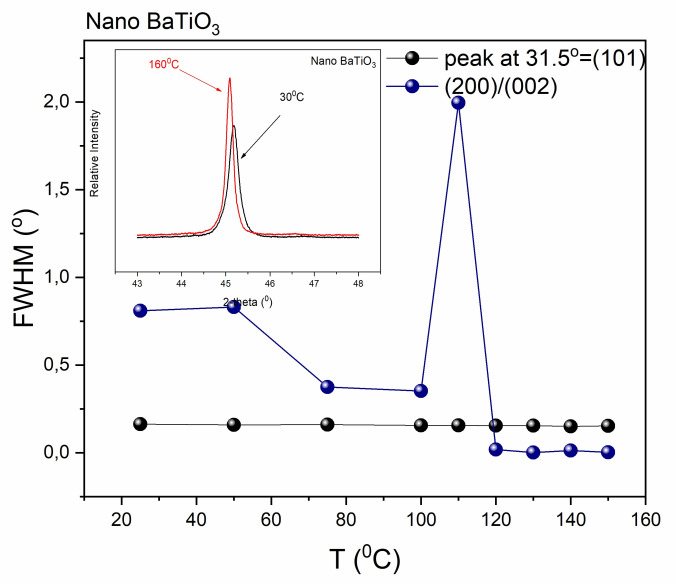
FWHM as a function of temperature for the diffraction peak at 31.5° (101) and for the FWHM ratio of (200)/(002) diffraction peaks. Inset shows the XRD patterns of nano-BT particles at 30 °C and 160 °C respectively. Lines are visual aid.

XRD patterns of nano-BT particles deviate from the corresponding ones of both tetragonal and cubic phase, Figure 5b. From the two-diffraction peaks, being the “trademark” of the tetragonal phase in micro-BT particles, only the (200) one is recorded, indicating the absence or the decrement of the tetragonal phase. However, the remaining peak is not identical to the fingerprint of the pure cubic phase. It appears as a broader and less-symmetric peak, which is considered as an indication for the co-existence of both tetragonal and cubic lattices at temperatures below T_c_. As the temperature increases this peak becomes sharper approaching the foretype of the cubic phase. The coexistence of both tetragonal and cubic phase, below T_c_, in nano-BT particles becomes evident from the variation of Full Width at Half Maximum (FWHM) with temperature. Figure 6 depicts the FWHM ratio of the (200)/(002) peaks, indicative of the tetragonal phase, as calculated by performing deconvolution at XRD patterns of the nano-BT particles.

The FWHM peak at 31.5 ° 2-theta is used as a reference, in order to ensure that there is no impact of the temperature on the non-related to the transition peaks. The formed peak at 115 °C in Figure 6 is attributed to the structural transition in BaTiO_3_ nanoparticles and indicates indirectly the critical temperature. The relocation of the peak, related to the transition, at lower temperatures is attributed to the polycrystalline character of the nanopowder. Decreasing the particle size, the phase transition has a more diffuse character displaying a lower transition enthalpy.

**Figure 7:**
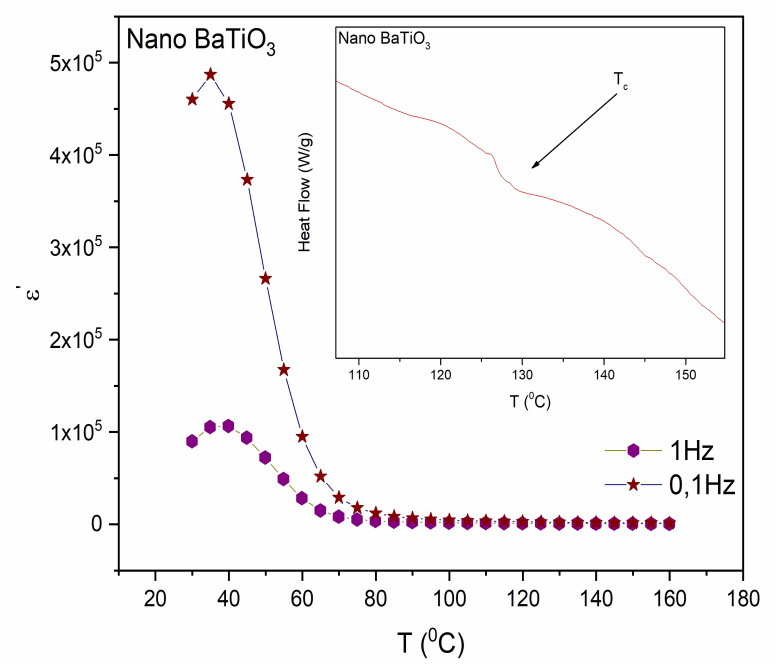
Real part of dielectric permittivity for nano-BT particles as a function of temperature at 1 and 0.1 Hz. Inset shows the DSC thermograph of nano-BT particles with 30 °C/min heating rate, endo down.

Furthermore, electrical characterization was also performed on nano-BT particles for the detection of the structural transitions. Spectra of the real part of the dielectric permittivity $\left(\varepsilon^{\prime }\right)$ as a function of temperature at 0.1 Hz and 1 Hz in nano-BT particles are shown in Figure 7. The critical temperatures for these transitions can be detected by the formed peaks in the real part of dielectric permittivity versus temperature spectra. Interestingly, the t→c transition is not evident in nanoparticles’ spectra as opposed to micro-BT particles (Figure 4a), indicating that the tetragonal phase is not present in the nano-BaTiO_3_ particles or its extent is significantly reduced. At lower temperatures, just as in the case of micro-BT, nanoparticles’ spectra form an intense peak at 40 °C, which corresponds to the transition from the orthorhombic (o→t) to the tetragonal phase of nano-BT particles. By these means, tetragonal phase should exist in BT nanoparticles above 40 °C, although not evidently change to cubic at *T_C_*. As mentioned previously BT’s tetragonality diminishes or either vanishes with the reduction of particles’ dimensions, since the value of the aspect ratio c/a approaches unity. Possibly, dielectric spectroscopy has not the required sensitivity to record this structural variation when the number of the entities undergoing the transition is limited and the variation of polarization is low. Interestingly, the values of the real part of dielectric permittivity for BT nanoparticles appear to be significantly higher than the corresponding ones of microparticles. The latter should be attributed to the increased interfacial area between grains in the case of nanoparticles, which result to enhanced interfacial polarization and permittivity at low frequencies.

Likewise, in micro-BT particles, the high heating rate in the DSC thermograph reveals a t→c transition at approximately 125 °C. First order transitions can be identified with a high heating rate rather than with a low one, because at a low heating rate the molecule obtains sufficient time to transit to intermediate pseudo-static phases and as a result the transition could not be detectable.

Decreasing the crystallite size in fine BT powder in the sub-micron range results in a decrement of the crystals tetragonality, that is the dimensional ratio c/a of the unit cell approaches unity [10]. The progressive reduction of tetragonality leads to the stabilization, at room temperature, of the paraelectric-cubic phase. Although it is not indisputably determined, studies have shown that powders with crystal size below 50 nm do not exhibit tetragonal structure at room temperature [15,38]. As a consequence, T_c_ is related to the crystallite size. In the case of a powder with fine nanoparticles displaying a distribution of sizes, tetragonality of each size corresponds to a specific critical temperature and the overall effect is a dispersion of the ferroelectric to paraelectric transition over a temperature range [10,11]. At this point it should be recalled that, according to the supplier’s data sheet and data from Figure 1a, the employed nanoparticles are smaller than 100 nm, with varying sizes.

**Figure 8:**
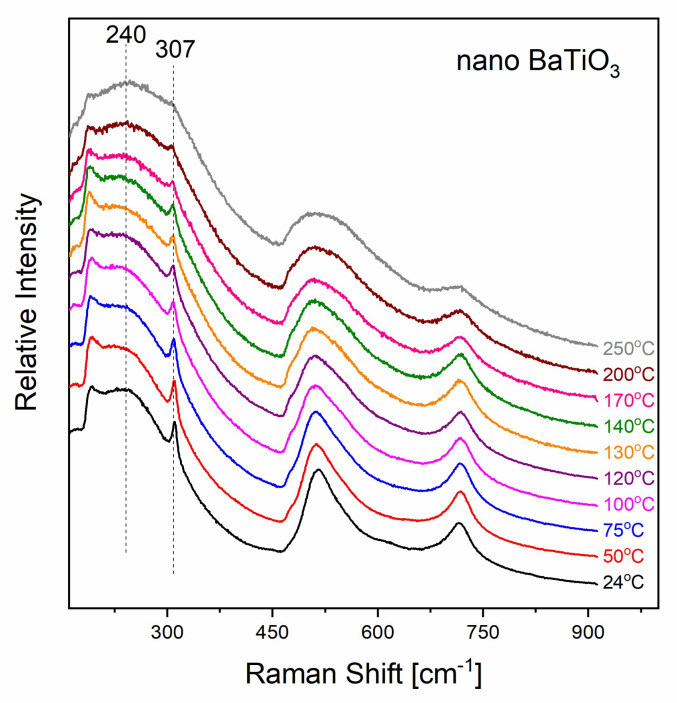
Raman spectra of nano-BT particles in the temperature range from 24 °C to 250 °C.

Similar to the case of BT micro-sized particles, the Raman spectrum, of BT nanoparticles at ambient temperature (Figure 8), possesses bands characteristic of the tetragonal BT phase. The sharp bands around 307 cm^−1^ and 718 cm^−1^ are assigned to the ferroelectric tetragonal phase. The abrupt lessening at 185 cm^−1^ is attributed to the interference between Raman scattering form of two vibrations modes with overlapping frequency range and is considered as a typical spectral feature of nanoparticles [31,39,40,41]. The spectral parameters of the characteristic sharp Raman band at 307 cm^−1^, associated with the tetragonal phase, differ from the corresponding ones for the micro-sized BT. More specifically, the FWHM of the peak is considerably greater while its integrated intensity is lower. Moreover, there are differences in the temperature dependence of these particular spectral features between micro- and nano-sized samples.

**Figure 9:**
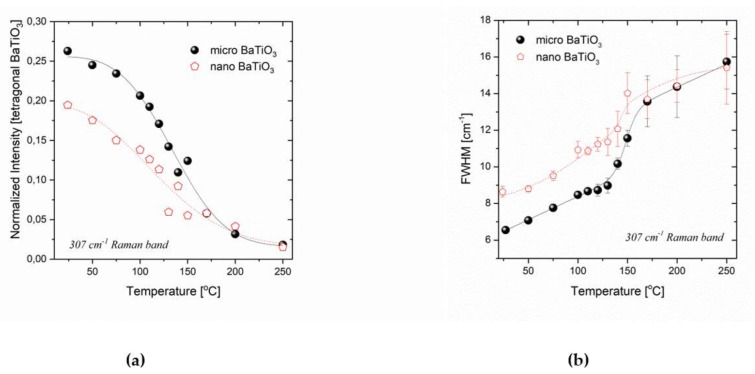
Normalized intensity (**a**) and FWHM of the 307cm^−1^ band (**b**) as a function of temperature, for the micro- and nano-sized BaTiO_3_ particles.

Following the analysis of Smith et al. [35] we monitored the phase transition of BaTiO_3_ in the temperature dependent Raman spectra (up to 250 °C). The full width at half maximum (FWHM) of the vibrational mode at 307 cm^−1^, characteristic of the tetragonal phase, as well as its corresponding integrated intensity varies as a function of temperature. More specifically in the transition temperature region the normalized intensity of this band decreases, while its width increases as temperature is raised. The temperature dependence for both peak’s characteristics differs for the micro- and nano-shaped BaTiO_3_ samples (Figure 9). The normalized intensity is the integrated intensity ratio of the 307 cm^−1^ band to the ~250 cm^−1^ band in accordance with [35]. The width of the peak was extracted after fitting the respective spectral region with a Lorentzian line profile. Notice the prominent error bars at high temperatures (plot of the width of the peak as a function of temperature); they result from the corresponding low signal/intensity of the band in this temperature region. Although the general trends signifying the tetragonal to cubic transition are similar, i.e., the intensity of the band decreases with temperature down to practically zero values above 250 °C and the width of the peak increases with temperature with a weak jump at ~145 °C, the breadth and strength of the transition is found to be altered for the nano-sized system. In general, the phase transition is dispersed over a considerable range of temperatures for the nanoparticle case if compared to the microparticle one.

**Figure 10:**
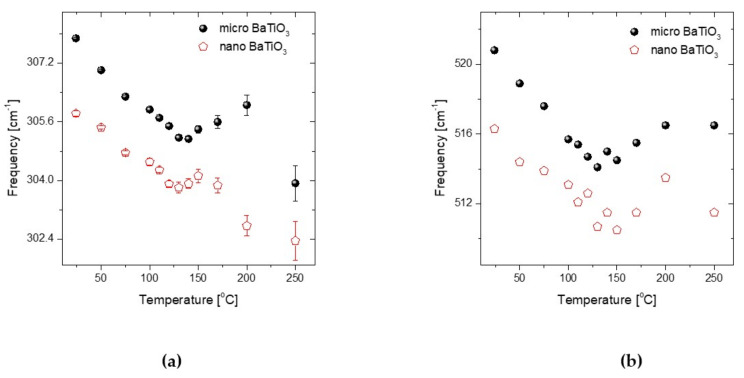
Temperature dependence of the (**a**) ~307 cm^−1^ and (**b**) ~520 cm^−1^ bands for the nano- and micro-sized BaTiO_3_.

The temperature dependence of additional spectral features in the Raman spectra of micro- and nano-sized BaTiO_3_ may be also used as indicators of the transition. For instance the frequency of the ~307 cm^−1^ and ~520 cm^−1^ band is given for temperatures ranging from ambient up to 250 °C in Figure 10. Obviously the Raman shift of the bands for both micro- and nano-sized BaTiO_3_ forms a minimum in the vicinity of the transition temperature. The corresponding temperature dependence of the FWHM and intensity for the 520 cm^−1^ band, together with the respective dependence of the spectral features of the ~715cm^−1^ band are given in Figures S2 and S3 of the Supplementary Materials.

### 3.3. BaTiO_3_ Nanocomposites

**Figure 11:**
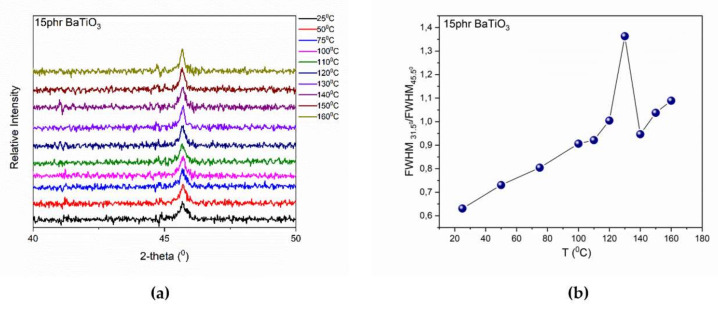
(**a**) XRD patterns in the temperature range from 25 °C to 160 °C and (**b**) FWHM_31.5_°/FWHM_45.5_° as function of temperature for the 15 phr BaTiO_3_/epoxy nanocomposite. Line in Figure 10b is visual aid.

Finally, extensive analysis was performed in order to identify the structural t→c transition in the case of the 15 phr BaTiO_3_/epoxy nanocomposite. According to the analysis conducted previously, the transition is detectable through XRD patterns. Therefore, XRD studies were performed in the 15phr nanocomposite in a wide temperature range (25 °C to 160 °C). XRD patterns exhibit experimental noise due to the presence of the amorphous epoxy resin. Figure 11a shows that the peak corresponding to the transition is broader and non-symmetrical at low temperature.

However, as the temperature is increased this peak is getting sharper and more intense, indicating the t→c transition. The variation of the FWHM upon temperature was employed as an indicator of the recorded transition. Since it was not possible to perform the deconvolution process for the two peaks (002) and (200), due to the presence of the amorphous phase of the polymer matrix, it was preferred to employ as an indicator factor of the t→c transition the FWHM ratio of the peaks at 31.5° and 45.5°. The peak at 31.5° as it has been already mentioned is temperature independent (Figure 5) and the peak at 45.5° is related to the transition. In Figure 11b, the formation of a peak at approximately 130 °C is evident, which reflects the t→c transition of nano-BT particles in the 15 phr BaTiO_3_/epoxy nanocomposite.

## 4. Conclusions

Ferroelectric materials have aroused a lot of scientific interest since their properties have a direct influence in their potential applicability. In this study, the ferroelectric to paraelectric transition from the tetragonal to cubic crystal phase in BT has been examined. The dielectric properties of BT change sharply between the two crystal structures. Moreover, extensive analysis of the structural transitions in micro- and nano-BT particles was performed along with the respective analysis in the 15phr BT/epoxy nanocomposite system. In the case of micro- and nano-BT particles the t→c transition was determined via the variation with temperature of the FWHM ratio of the (200) and (002) diffraction peaks. The resulting peak formation at approximately 120 °C, in both cases, is indicative of the t→c transition and it reflects the discontinuous change of polarization. Furthermore, DSC, BDS and LRS data gave evidence of the t→c transition, while dielectric spectroscopy was able to determine the o→t transition in both micro- and nano-BT particles. Below critical temperature (*T_C_*), it was found that tetragonal and cubic phase co-exist in nano-BT particles. Finally, XRD patterns of the 15phr BT/epoxy nanocomposite reveal indications of the t→c transition, via the variation of the FWHM ratio of the peaks at 31.5 ° and 45.5 ° upon temperature. The present study investigates the ferroelectric to paraelectric transition of nano-BT crystals, and its size dependence, via several experimental techniques. This contributes in raising disputes concerning this transition. Moreover, provides evidence for the dielectric properties of both micro- and nano-BT particles at the critical points of their transitions. Finally, a qualitative tool for the determination and analysis of this transition is proposed.

## Supplementary Materials

The following are available online, Figure S1: Representative fitting figures for the deconvolution process in micro-BT particle with peak-o-mat software at (a) 75 °C, (b) 120 °C and (c) 140 °C, Figure S2: The temperature dependence of (a) the FWHM and (b) the absolute intensity for the 520 cm^−1^ band. This band is composed of several peaks some of which are attributed to the tetragonal phase while a broad one (~300 cm^−1^) survives above the critical temperature, Figure S3: The temperature dependence of (a) the frequency, (b) the FWHM and (c) the absolute intensity for the 715 cm^−1^ band. For the case of the micro-sized system intensity of the 715 cm^−1^ band almost vanish at high temperatures. This behavior does not match the corresponding one of the nano-sized system. For this reason the error bars in this temperature region is considerably high for micro-BaTiO_3_.
