# Supplementary material for: On the Ferroelectric to Paraelectric Structural Transition of BaTiO3 Micro-/Nanoparticles and Their Epoxy Nanocomposites
Source: Molecules. 2020 Jun 9;25(11):2686. doi: 10.3390/molecules25112686 (PMC7321176; doi:10.3390/molecules25112686)
Supplement: Supplementary file 1 [file molecules-25-02686-s001.pdf]

Supplementary Materials

On the Ferroelectric to Paraelectric Structural Transition of $BaTiO_3$ Micro-/Nanoparticles and Their Epoxy Nanocomposites

Georgia C. Manika [1,2], Konstantinos S. Andrikopoulos [3] and Georgios C. Psarras [1,*]

1 Smart Materials & Nanodielectrics Laboratory, Department of Materials Science, University of Patras, 26504 Patras, Greece
2 Department of Industrial and Materials Science, Division of Engineering Materials, Chalmers University of Technology, SE-41296 Gothenburg, Sweden; georgia.manika@chalmers.se
3 Foundation for Research & Technology-Hellas (FORTH), Institute of Chemical Engineering Sciences (ICE-HT), 26504 Patras, Greece; candrik@iceht.forth.gr
* Correspondence: G.C.Psarras@upatras.gr; Tel.: +302610996316

(A). Deconvolution of the XRD spectra

In the case of micro- and nano-BT particles, deconvolution was conducted by keeping the location of two peaks constrained and fitting the XRD spectra with two peaks varying their intensity and width. The aim was to reveal a critical point (local maximum) at, or very close to, T_c. Figure S1 shows three representative fittings. In the case of the nanocomposite, due to the noisy spectra related to the amorphous polymer matrix, deconvolution was conducted by employing the temperature independent peak at 31.5°.

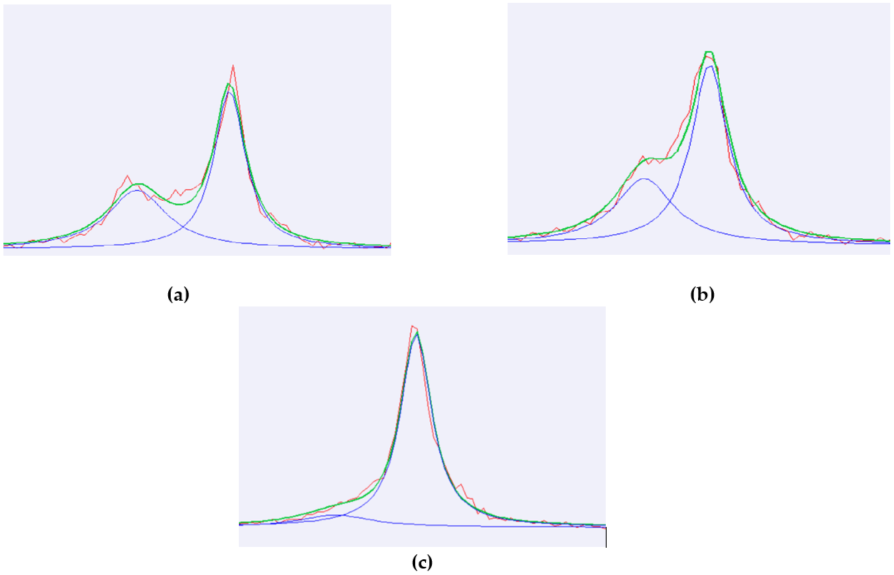

Figure 1. Representative fitting figures for the deconvolution process in micro-BT particle with peak-o-mat software at (a) 75°C, (b) 120°C and (c) 140°C.

(B) Raman spectra

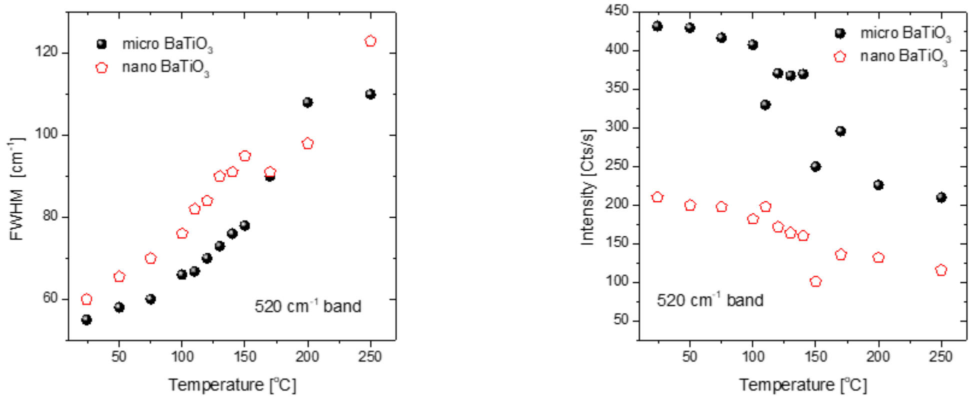

(a) **(b)**

Figure 2. The temperature dependence of (a) the FWHM and (b) the absolute intensity for the 520 cm^{-1} band. This band is composed of several peaks some of which are attributed to the tetragonal phase while a broad one (~300 cm^{-1}) survives above the critical temperature.

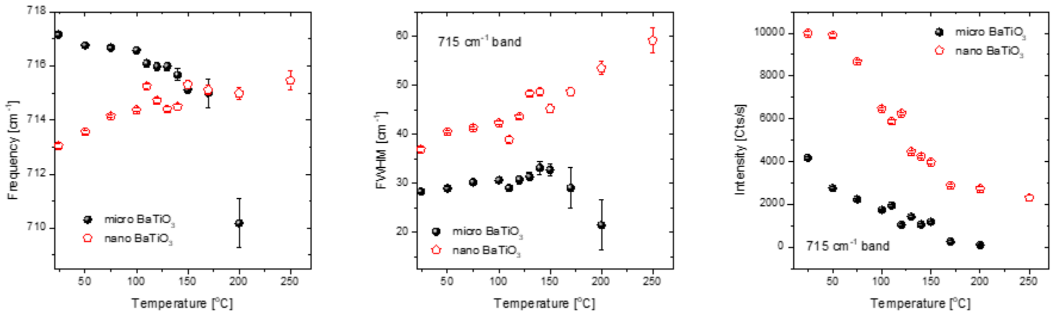

(a) (b) (c)

Figure 3. The temperature dependence of (a) the frequency, (b) the FWHM and (c) the absolute intensity for the 715 cm^{-1} band. For the case of the micro-sized system intensity of the 715 cm^{-1} band almost vanish at high temperatures. This behavior does not match the corresponding one of the nano-sized system. For this reason the error bars in this temperature region is considerably high for micro-$BaTiO_3$.
